# Paper-based biosensors as point-of-care diagnostic devices for the detection of cancers: a review of innovative techniques and clinical applications

**DOI:** 10.3389/fonc.2023.1131435

**Published:** 2023-06-30

**Authors:** Mehrdad Mahmoodpour, Bahman Abedi Kiasari, Merat Karimi, Arezou Abroshan, Danial Shamshirian, Hamed Hosseinalizadeh, Alireza Delavari, Hamed Mirzei

**Affiliations:** ^1^ Department of Medical Biotechnology, Faculty of Paramedicine, Guilan University of Medical Sciences, Rasht, Iran; ^2^ Virology Department, Faculty of Veterinary, The University of Tehran, Tehran, Iran; ^3^ Institute of Nanoscience and Nanotechnology, University of Kashan, Kashan, Iran; ^4^ Student Research Committee, Faculty of Veterinary Medicine, Shahid Bahonar University, Kerman, Iran; ^5^ Chronic Respiratory Diseases Research Center, National Research Institute of Tuberculosis and Lung Diseases (NRITLD), Shahid Beheshti University of Medical Sciences, Tehran, Iran; ^6^ Student's Scientific Research Center, Tehran University of Medical Sciences, Tehran, Iran; ^7^ Research Center for Biochemistry and Nutrition in Metabolic Diseases, Institute for Basic Sciences, Kashan University of Medical Sciences, Kashan, Iran

**Keywords:** paper-based devices, biosensors, early diagnosis, dipsticks, lateral flow assay, microfluidic paper-based device

## Abstract

The development and rapid progression of cancer are major social problems. Medical diagnostic techniques and smooth clinical care of cancer are new necessities that must be supported by innovative diagnostic methods and technologies. Current molecular diagnostic tools based on the detection of blood protein markers are the most common tools for cancer diagnosis. Biosensors have already proven to be a cost-effective and accessible diagnostic tool that can be used where conventional laboratory methods are not readily available. Paper-based biosensors offer a new look at the world of analytical techniques by overcoming limitations through the creation of a simple device with significant advantages such as adaptability, biocompatibility, biodegradability, ease of use, large surface-to-volume ratio, and cost-effectiveness. In this review, we covered the characteristics of exosomes and their role in tumor growth and clinical diagnosis, followed by a discussion of various paper-based biosensors for exosome detection, such as dipsticks, lateral flow assays (LFA), and microfluidic paper-based devices (µPADs). We also discussed the various clinical studies on paper-based biosensors for exosome detection.

## 1 Introduction

With the deterioration of the human living environment, cancer poses a serious threat to human life and health and has become a global public health problem (1). Cancer has long been the leading cause of death worldwide (2). Millions of people die from cancer every year, most of them in developing countries (3). Early detection and successful therapy are two of the major difficulties in the fight against cancer (4, 5). Therefore, early cancer detection has long been considered an important component of screening and treatment (2). All attempts to achieve this goal depend on recognizing cancer markers, which can be found in various forms in tumor tissue and patients’ blood, such as DNA, miRNA, and proteins (6).

Tumor marker detection technology can be known as an important tool and strategy for detecting several cancers (7). Exosomes are nanoscale (30-150 nm) extracellular vesicles formed by a variety of cells, including immune cells, neurons, and tumor cells, and were identified by Johnstone in 1987 (8). Exosomes are critical for intercellular communication. They transport large amounts of molecular data, such as nucleic acids and proteins, which are subsequently taken up by surrounding cells or transferred to the blood and eventually taken up by distant cells. Cancer-associated exosomes are nanoscale membrane vesicles that perform important functions in the tumor microenvironment (TME) (2, 9). According to recent data, exosomes can carry specific payloads such as proteins and nucleic acids that reflect tumor status (2). Remarkably, the molecular information on the surface of exosomes can indicate the origin of cancer (9). As a result, exosomes are increasingly used as indicators for benign tumor detection (10, 11). Exosomes have been mentioned in the field of liquid biopsy as a potential noninvasive biomarker for various diseases, especially autoimmune diseases and cancer, because they are present in greater quantities in the bloodstream (2, 12, 13). However, the detection and analysis of exosomes are difficult due to the lack of practical and efficient methods. The development of non-invasive technologies to detect tumor markers is the research topic for earlier cancer diagnosis (7). Over the years, various methods such as mass spectrometry, western blotting, flow cytometry, and enzyme immunoassay have been developed to study exosomes in biological fluids such as blood, urine, breast milk, and saliva (14). Although these methods are successful and reliable, they have several drawbacks, such as the need for expensive equipment, and time-consuming technical steps, which have limited their widespread use (15, 16). The need for continuous real-time monitoring of exosomes and bridging the gap between the availability of healthcare facilities and demand has led to the adoption of innovative techniques such as paper-based biosensors, especially in developing countries where healthcare spending is limited (7). Given the importance of the topic, in this review, we aim to analyze paper-based biosensors as one of these platforms for cancer exosome detection.

## 2 Exosomes

Extracellular vehicles (EVs) are transportable vesicles that enable cells to exchange biological substances (17). Several subclasses of EVs have been postulated, such as ectosomes, microvesicles, microparticles, oncosomes, apoptotic bodies, exosomes, etc (18). Exosomes are extracellular vesicles ranging in size from 30 to 150 nm that contain lipids, proteins, and nucleic acids (19). When examined under the electron microscope, exosomes often have a “cup-shaped” or “saucer-like” appearance (20). Cryogenic electron microscopy is commonly used to study the spherical shape of exosomes. They have a symmetrical phospholipid membrane topology, can be released from various cell types, and are detected in many body fluids, including blood, urine, and saliva (7). Exosomes can now be extracted from almost all cell types and various physiological and pathological secretions such as blood, mucus, breast milk, urine, cerebrospinal fluid, and fluid surrounding the lungs (21, 22). Many cell types release exosomes in both healthy and pathological situations (20). Exosomes are released from a variety of human cell types, but their lipid bilayers envelop only a tiny fraction of the cytoplasm of their parent cells and lack cell organelles (17). The composition of the exosomes reflects the health and pathology of their mother cells and is linked to their environmental stimulation (23). Furthermore, the different compositions of exosomes strongly depend on their initial cell types and activities, implying that exosomes have cargo selectivity (24).

**Figure 1:**
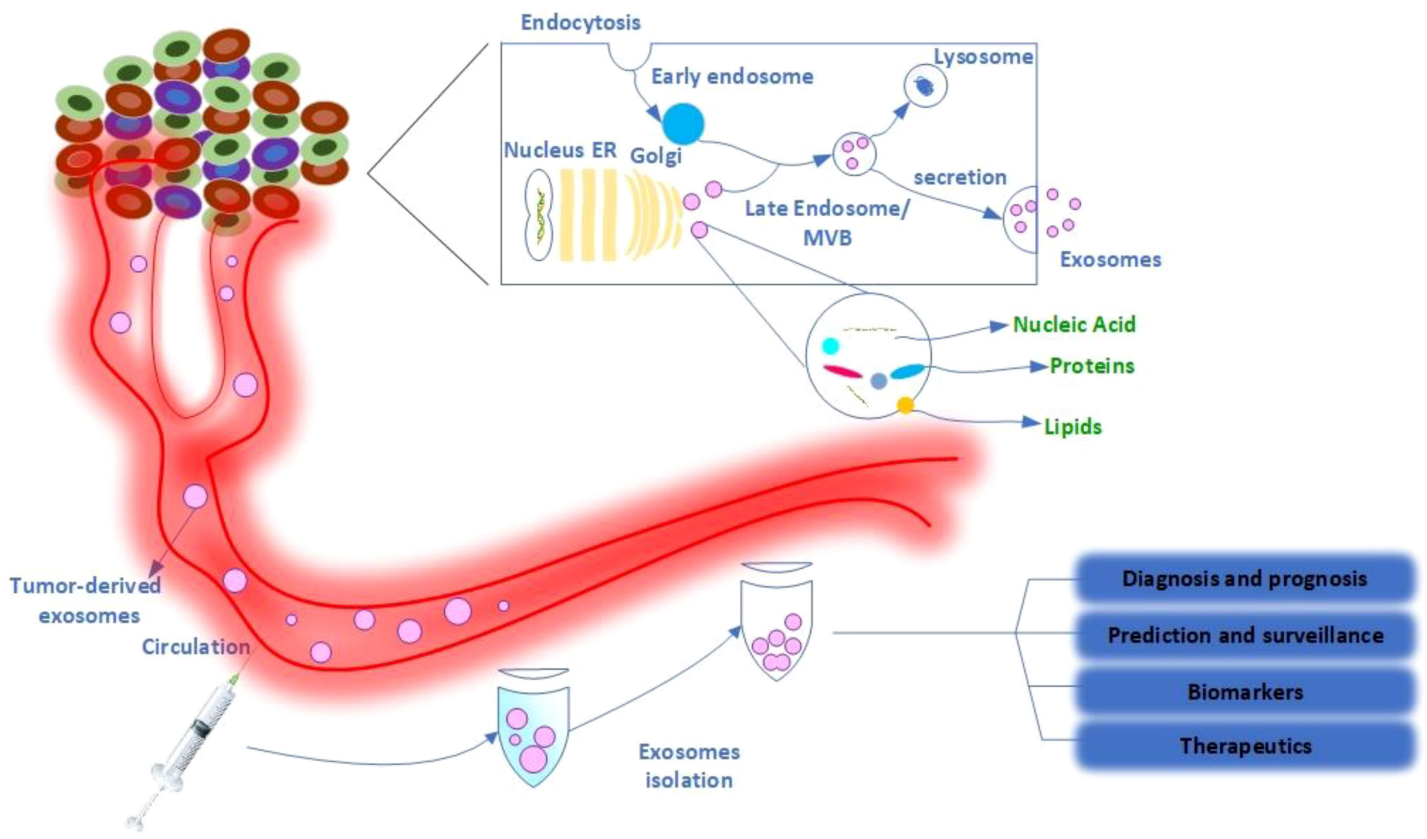
Schematic representation of exosome production, secretion, and transfer of cargo from donor cancer cells to the bloodstream and its therapeutic application. Exosomes are a type of extracellular vesicle released by both normal and cancer cells. Internal vesicles are formed by inward budding of the cellular compartments of the multivesicular body (MVB). When the MVBs fuse with the plasma membrane, these intracellular vesicles are released as exosomes that carry organic compounds specific to cancer cells, such as DNA, RNA, and proteins, into the various biological fluids, including serum and urine of cancer patients. Exosomes in the bloodstream have several biomedical applications, including diagnostic and prognostic applications, predictive and surveillance applications, and therapeutic applications such as drug delivery.

Exosome synthesis (**Figure 1**) begins when the cell membrane is internalized to generate an early endosome (19). The early endosomes mature into late endosomes or multivesicular bodies (MVBs) densely packed with intraluminal vesicles (ILVs). The MVB binds to the plasma membrane and releases ILVs into the extracellular environment. These ILVs can also be transported to lysosomes and degraded (2). In this process, the machinery of the endosomal sorting complex required for transport (ESCRT) is critical (2). Members of the ESCRT family, the tumor susceptibility gene 101 (TSG101)/VPS23 and the ALG-2-interacting protein X (Alix), also known as AIP1, are involved and have recently been identified in the literature as important exosome components (25). The Rab family of small GTPase proteins, particularly Rab27a and Rab27b, controls this process (26, 27). However, the mechanism by which exosomes are produced and secreted remains largely unclear due to differences between cell types and their status (2). Exosomes consist of a distinct lipid bilayer with an average diameter of 5 nm. Cholesterol, phosphatidylserine, and ceramide are abundant in the lipid bilayer (3). Consistent with donor cell membranes, constitutive membranes consist of tetraspanins, adhesion molecules, proteases, and transmembrane receptors. Exosomes have a variety of molecular components that change depending on their origin and state (28, 29). Exosomes contain the unique proteins commonly used as markers to confirm the presence of these vesicles, such as TSG101, Alix, CD63, CD81, and Rab family members (2). Exosomes also contain a large amount of mRNA, DNA, microRNA (miRNA), long non-coding RNA (LncRNA), and other nucleic acid families (2, 30, 31). In addition, lipids such as cholesterol, phospholipids, glycerophospholipids, and sphingolipids are important components of exosomes (2). Cholesterol is a waxy, fat-like material that is present in the lipid bilayer of exosomes and functions as an exosome biomarker (32). They build the structure of the bilayer membrane and keep it stable (33). Some active lipids (e.g., prostaglandins and leukotrienes) and enzymes related to lipid metabolism have also been observed in EVs (34, 35), suggesting that EVs may play a role in lipids associated with cancer progression. Notably, the molecular signature of tumor cells is increased in exosomes transferred between tumor cells and normal cells in malignancies (2).

## 3 Exosomes: a source of cancer biomarkers

Exosomes isolated from cancer patients could be used as biomarkers for cancer diagnosis, prognosis, prediction, monitoring, therapeutic targets, and even anticancer drug carriers (**Figure 1**) (36, 37). Exosomes, the most common type of micro vesicles, are both stable and numerous in human fluids (> 10^9^ vesicles/ml in the blood) (38). Exosomes are secreted by cancer cells in more significant quantities than by healthy cells, resulting in a large quantity of circulating exosomes (2). Tumor-derived exosomes typically contain tumor antigens as well as immunosuppressive proteins such as FasL (Fas ligand)/CD95L, TRAIL (TNF-related apoptosis-inducing ligand)/TNFSF10, and TGF-ß (transforming growth factor ß) (39, 40). Tumor-derived exosomes play a critical role in tumor development, metabolism, and migration (41). Exosomes released from CXCR4 (CXC motif chemokine receptor 4)/CD184-overexpressing breast cancer cells contained significant amounts of stemness-related markers and metastasis-related messenger ribonucleic acids (mRNAs) (42). Exosomes produced by CD184-overexpressing cells exhibited significant expression of stemness-related markers and increased invasiveness and metastatic potential of cancer cells (42). Similarly, exosomes produced by adipose-derived mesenchymal stem cells promoted cancer migration and proliferation dependent on the Wnt/ß-catenin signaling pathway (43). In colorectal cancer (CRC), exosomes from cancer-associated fibroblasts have been shown to activate cancer stem cells and contribute to radio resistance and chemoresistance *via* the Wnt signaling pathway (44). Moreover, the chemotherapeutic agent gemcitabine/Gemzar/2′,2′-difluorodeoxycytidine increased the expression and secretion of miR-146a/MicroRNA 146a and SNAI1/Snail in exosomes derived from cancer-associated fibroblasts, allowing pancreatic cancer recipient cells to proliferate and develop drug resistance (45). Reducing exosome production by inactivating neutral sphingomyelinase (SMase) during gemcitabine therapy dramatically decreased the survival rate of co-cultured pancreatic cancer cells (45).

EVs carrying various molecular cargoes, including nucleic acids (DNAs, mRNAs, microRNAs, long noncoding RNAs, and various noncoding RNAs), polypeptides, lipids, and metabolites, are actively taken up by target cells, resulting in structural and physiological changes (23, 46). Transfer of oncogenic payloads *via* EVs and tumor-derived exosomes has been shown to enhance oncogenic signaling pathways associated with cancer development and the TME (47, 48). Several studies have shown that the majority of exosomal DNAs (exoDNAs) in tumor-derived exosomes are dsDNA (double-stranded DNA) from all chromosomes (31). The miRNAs identified in tumor-derived EVs influence the translational patterns of recipient cells during tumor development (49). Transmission of miRNAs (oncomiRs) *via* EVs enhances cell interactions in normal and pathological processes (17). Encapsulation of miRNAs in exosomes restricts miRNA degradation, which increases the diagnostic value of exosomal miRNAs in cancer diagnosis (50). It is now known that many key proteins are not spontaneously but selectively packaged into EVs. Integrins are a large class of cell surface receptors that control reversible signaling between the interior and exterior of a cell (17). In turn, exosomal integrins play an important role in directing exosomes to target cells for specialized and effective signal transduction (51, 52), enabling specific and efficient intercellular communication (35).

## 4 Exosomes in cancer progression

Tumor-derived EVs with protumorigenic activity promote cancer aggressiveness, distant metastasis, extracellular matrix alteration, angiogenesis, therapy resistance, and immunosuppression (23, 46), indicating the importance of tumor-derived EVs in cancer development. The transfer of metastatic elements (i.e., oncogenic proteins or oncogenic microRNAs, oncomiRs) has the potential to activate and alter signaling cascades, morphologies, and activities in target cells (48, 53). For example, exosomal cluster of differentiation 44 (CD44)/HCAM is transferred from ovarian cancer cells to peritoneal mesothelial cells, resulting in peritoneal mesothelial cells with a mesenchymal and spindle-shaped morphology and promoting cancer invasion (54). Several studies have linked exosomes to epithelial-mesenchymal transition (EMT) during cancer growth (55). The uptake of exosomes by pancreatic cancer Kupffer cells/stellate macrophages cells increased the establishment of premetastatic niches by enhancing TGF-ß production and fibronectin expression by hepatic stellate cells, thus promoting liver metastasis (56, 57). Moreover, tumor-derived exosomal miR-1247-3p was shown to stimulate the ß1-integrin/NF-кB (nuclear factor ‘kappa-light-chain-enhancer’ of activated B-cells) signaling axis, leading to the recruitment of cancer-associated fibroblasts and promoting liver cancer metastasis to the lung (53).

Exosomes are a multifunctional regulator of cancer development (2). They usually contain cancer-related proteins in various malignant tumors and are able to alter the TME to affect neighboring cells or cells at specific distant sites (2). Therefore, they can serve as a bridge between normal and malignant cells and facilitate cancer diagnosis (58). Exosomes play a critical role in tumor development and metastasis. Exosomes can transport growth-promoting genes to promote the formation of metastatic cancer cells (2). Exosomes carrying epidermal growth factor receptor (EGFR) released by GC(gastric cancer) cells promote the formation of liver-specific metastases (59). The transported EGFR was found to efficiently activate hepatocyte growth factor (HGF)/scatter factor (SF) by suppressing the expression of MiR-26a/miR-26b (60). Moreover, the enhanced paracrine coupling of SF to the c-MET receptor on migrating cancer cells creates a fertile “soil” for the “seeds” that promote metastatic cancer cell implantation and proliferation. Thus, EGFR-containing exosomes secreted by cancer cells could support the formation of a liver-like microenvironment that enables liver-specific metastasis (59). It has also been shown that exosomal miRNAs are able to manipulate the transcriptome pool of target cells that promote tumorigenesis in non-tumorigenic epithelial cells (61). Recent research has shown that exosomal microRNAs play a critical role in carcinogenesis. Exosomal miRNAs have been found to serve as essential mediators of cell-cell communication, act as autocrine, paracrine, and endocrine signaling regulators, and have the ability to regulate gene expression. Exosomal microRNAs are very stable and easily detectable in physiological fluids, suggesting that miRNAs could be used as diagnostic and prognostic cancer biomarkers (62). Exosomal miRNAs can provide information about the cell type from which they are released, the target, and the cellular state, including treatment resistance. Valadi et al. showed that exosomes can contain greater amounts of miRNAs such as let-7, miR-1, miR-181, and miR-375 than the cells of origin, indicating preferential packing of miRNAs in exosomes (62, 63). Pigati et al. also found that certain miRNAs released by breast cancer cells, such as miR-451 and miR-1246, are secreted regardless of their internal concentration, whereas most miRNAs released by non-malignant cells remain in the cells (64). In addition, exosomes can promote neoplastic proliferation or metastasis *via* bypassing cancer suppressors (2). Zhang et al. showed that parental tumor cells with typical PTEN (phosphatase and tensin homolog) activity decreased PTEN activity after spreading to the brain but not to other organs. After leaving the brain microenvironment, PTEN levels recover in metastatic tumor cells with PTEN loss in the brain. The downregulation of PTEN mRNA and PTEN protein in the brain is mediated by astrocyte-derived exosomes, which facilitate intercellular transfer of PTEN-targeted miRNAs to metastatic tumor cells, leading to PTEN depletion in the brain and promoting the development of brain metastases (65). In contrast to previous data reducing PTEN tumor suppressor function, Putz et al. found that ubiquitinated PTEN, a tumor suppressor protein, could be directly released from cells by exosomes through a mechanism dependent on Ndfip1/N4WBP5 ubiquitination of Lys13 (K13). PTEN exhibits functional activity when internalized by recipient cells by reducing phosphatidylinositol 3-kinase (PI3K) signaling and decreasing cell proliferation. They showed that exosomal PTEN is an unexpected aspect of PTEN mobility that can extend its functionality beyond the cell boundary and is not restricted to one cell (66).

Cancer cells have also been shown to restrict glucose uptake by non-tumor cells to meet their own needs by secreting miRNA-122 highly expressed exosomes. MiRNA-122 inhibits the glycolytic enzyme pyruvate kinase (PKM)/PK and promotes metastasis by increasing the availability of nutrients in the pre-metastatic niche, while inhibition of miR-122 increases glucose uptake and reduces the incidence of metastasis. These results demonstrated that cancer-derived exosomes expressing miR-122 can alter systemic energy metabolism and thus promote disease progression (67). Exosomes can also induce angiogenesis in tumors and tumor-free tissues (68). However, there is limited information on the proangiogenic role of exosomes and their interaction with endothelial cells. In the study by Nazarenko et al., tumor-derived exosomes were found to contain Tspan8/tetraspanin 8, a highly conserved 4-transmembrane protein of the tetraspanin protein family, which could rapidly promote angiogenesis. They investigated the effects of exosomal Tspan8 on angiogenesis in a rat cancer model (69, 70). Internalization of Tspan8 and selective recruitment of proteins such as CD49d (the α chain of the CD49d/CD29 integrin heterodimer very late antigen 4 (VLA-4)), and CD106 by Tspan8 promoted vascular endothelial growth factor (VEGF)-independent regulation of numerous angiogenesis-related genes, including von Willebrand factor(VWF)/F8VWD, CXCL5, and Macrophage migration inhibitory factor (MIF)/glycosylation-inhibiting factor (GIF). Exosomes carrying the Tspan8-CD49d complex have been shown to increase EC growth, migration, budding, and maturation of EC progenitor cells (70, 70). Expressions of LINC00161/Linc-USP16 in exosomes derived from hepatocellular carcinoma (HCC) patient serum and HCC cell line supernatants were significantly associated with angiogenesis, metastasis, and poor survival (71). A mechanistic study revealed that Linc-USP16 directly targets miR-590-3p and activates the downstream Rho/ROCK signaling pathway. The ROCK pathway plays a critical role in regulating tumor cell angiogenesis and metastasis (71).

## 5 Application of exosomes as a liquid biopsy in clinical diagnosis

Traditional diagnostic methods for cancer rely primarily on endoscopy, computed tomography, X-rays, positron emission tomography, magnetic resonance imaging, and invasive tissue biopsies. These methods are neither accessible to large populations nor practical for repeated investigations (72). Recently, biomarker discovery by liquid biopsy, which enables noninvasive, rapid, dynamic, cost-effective, and accurate diagnosis for early cancer detection in real time, has emerged as an essential foundation of precision medicine (17). Several promising and useful biomarkers, such as circulating tumor cells (CTCs), cell-free DNAs (cfDNAs), and EVs, have been used for clinical detection in the last two decades to provide detailed information about cancer development thanks to rigorous research in liquid biopsy (31, 73, 74). Cancer cell-derived exosomes, generally 30-150 nm in diameter and 1.13-1.19 g/ml in density, are abundant and stable in body fluids (such as serum, plasma, and urine) (75). Oncologists first isolated cancer cell-derived exosomes from mice and patient blood, the most common sources of exosomes, and found that exosomes can represent the current state of tumor tissue. As a result, it was suggested that exosomes could be used as diagnostic and prognostic biomarkers for various malignancies (76). Platelet-derived extracellular vesicles (P-EVs) are the most abundant vesicles in human blood and account for two-thirds of all extracellular vesicles in peripheral blood (52). According to Liang et al. (2015), miR-223 is more abundant in platelet-derived extracellular vesicles from patients with hematogenous metastatic lung cancer than in healthy individuals (77).

**Figure 2:**
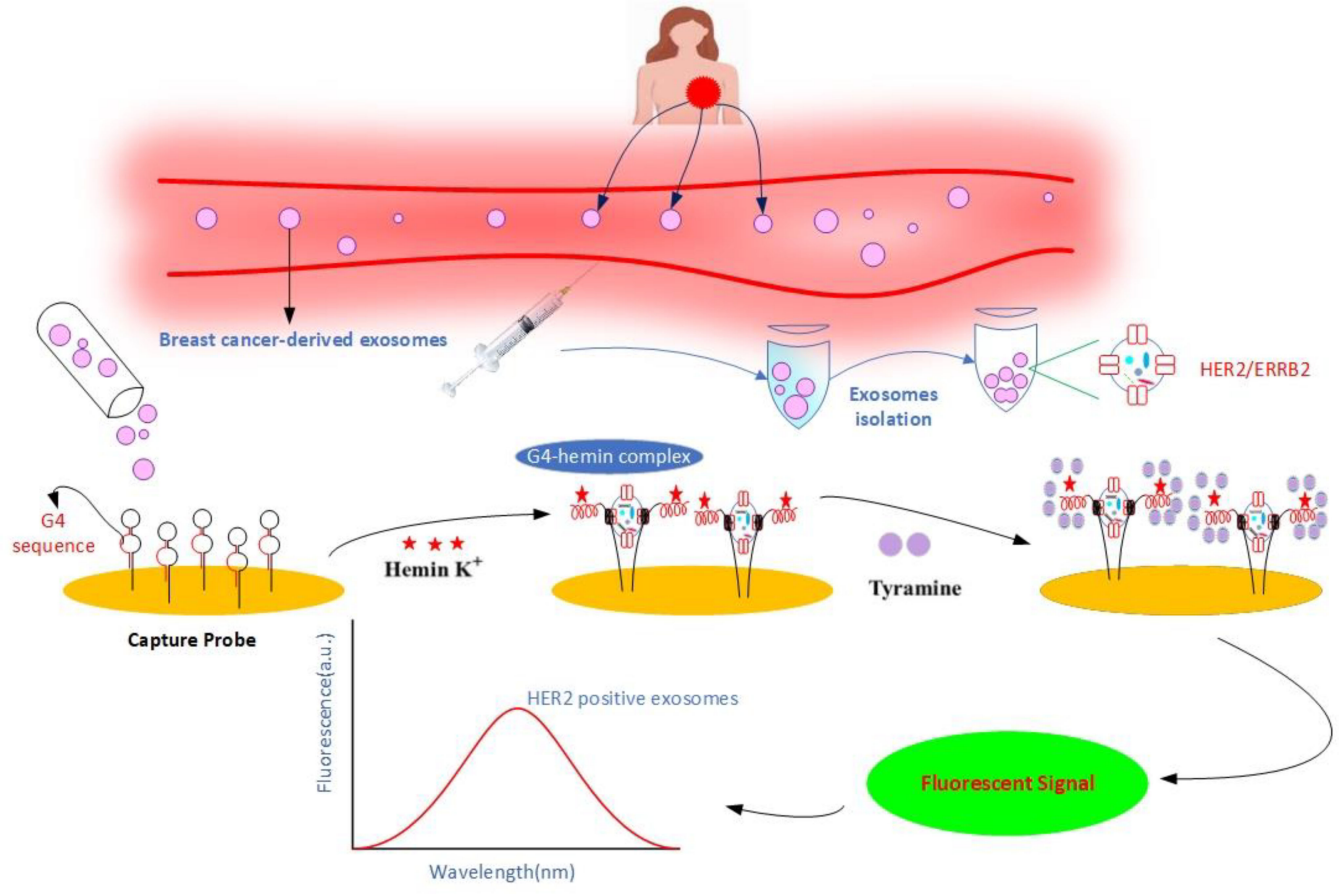
Schematic illustration of non-invasive detection of HER2-positive exosomes using a high-affinity capture probe and reformative tyramine signal amplification. HER2-positive exosomes play a crucial role in breast cancer diagnosis and therapy. First, exosomes are isolated from cell culture fluid or clinical samples and applied to the surface of a gold chip containing a capture probe. In the presence of HER2-positive exosomes, the capture probe binds to the HER2 protein and exposes the G4 sequence. The addition of hemin leads to the formation of a catalytically active G4-Hemin complex. In turn, the addition of tyramine promotes the catalysis of G4-Hemin, converting tyramine to a reactive oxidized intermediate with the help of H2O2, leading to the formation of a fluorescent signal. Exosomes are quantitatively detected by fluorescence intensity.

Exosomes have attracted much attention as researchers try to figure out what else they can do in cell biology and how they function in different organ systems. Several data series have shown that exosomes are involved in renal physiology and pathogenic processes of various renal diseases. For example, proteomic analysis of exosomes identified 295 proteins, including several protein products of genes already known to play a role in renal and systemic diseases. This suggests that exosomes are a potential source of biomarkers and pathogenic proteins for early diagnosis of renal cancer (78). McKiernan et al. investigated the efficacy of combining a urinary exosome gene expression test with standard of care (SOC) compared to SOC alone in prostate cancer (79). When combined with SOC, this novel test increased the sensitivity and accuracy of prostate cancer screening and diagnosis. In addition, it is possible that urine exosomes can be used for cost-effective cancer screening and clinical management (2). However, due to glomerular filtration, only exosomes of a certain size (100 nm) could be identified (80). Exosomes from breast milk play a unique role in cancer diagnosis for breast cancer patients (2). Qin et al. observed the expression of six breast cancer-associated proteins in exosomes from three milk samples (transition, maturation, and weaning milk) (81). They showed that exosomes from breast milk of healthy, breastfeeding women exhibited the most significant change in TGF-beta levels in weaning/early involution, resulting in changes in both benign and malignant breast epithelial cells associated with breast tumorigenesis and progression (81). Exosomes containing high levels of TGF2 induced EMT in both malignant and normal cells, as evidenced by (i) changes in cell shape, actin cytoskeleton, and loss of cell-cell junction structure, and (ii) enhancement of SMA and vimentin/VIM and reduction of E-cadherin/CDH1. Yu et al. reported that milk-derived exosomes have high expression of miR-29b and miR-21, which can trigger MAPK(mitogen-activated protein kinase)/ERK pathway and stimulate cell proliferation (82). The first cancer diagnosis based on exosomes was released in the USA on January 21, 2016 (83, 84). Although their therapeutic significance is still in its infancy, several researchers are interested in using cancer exosomes to monitor cancer progression, assess response to treatment, and predict prognosis (3). Cancer biomarkers are emerging as one of the most promising approaches for cancer diagnosis and treatment (3). A cancer biomarker is a ‘molecular signature’ that can offer precise information about cancer stages and the pathways leading to cancer development (85). Exosomes extracted from body fluids such as blood plasma, serum, and urine could be used to diagnose cancer at an early stage. Numerous studies have shown that exosomes released by cancer cells into the extracellular space can be used as biomarkers for the diagnosis of “various malignancies (20). Khan et al. observed surviving and its variants in serum exosomes and showed significantly higher levels of surviving in patients with early-stage breast cancer (86). Moon et al. demonstrated that exosomes containing fibronectin were significantly higher in early breast cancer patients, with a sensitivity of 65.1% and a specificity of 83.2% (87). According to Zhou et al., miR-105 is significantly expressed in bloodstream exosomes during the pre-metastatic or distant metastatic stages of breast cancer (88). They also found that this molecule and miR-181a were predominant in the serum exosomes of stage II and III breast cancer patients (88). Another study found that claudin/CLD proteins were expressed in the circulating vesicles of most ovarian cancer patients (89). Full-length claudins have been identified in the media as part of tiny lipid vesicles called exosomes released by ovarian cancer cells in culture. In addition, claudin-containing exosomes were found in 32 of 63 plasma samples from ovarian cancer patients. Only one of 50 samples from individuals who did not have cancer had claudin-positive exosomes. This suggests that the development of sensitive assays for the detection of claudins in blood may be critical as a screening biomarker for the detection of ovarian cancer (89). Isolation of human epidermal growth factor receptor 2 (HER2)-positive exosomes from the bloodstream has emerged as a novel method to improve breast cancer diagnosis and prognosis (90). Chen et al. developed an efficient fluorescent biosensor (**Figure 2**) for highly sensitive and selective detection of HER2-positive exosomes based on a high-affinity capture probe and reformative tyramine signal amplification (TSA) (90). The capture probes have a double-ring structure that is opened by the strong affinity between the probe and the overexpressed HER2 protein on the membrane of the exosomes, exposing their G-quadruplex DNA sequence (G4 DNA), while firmly capturing exosomes. The key steps in this approach are (1) extraction of exosomes (HER2 positive/negative) from breast cancer patients and (2) subsequent capture by the aptameric HER2 region of the capture probe anchored to the chip surface, and exposing the G4 DNA sequence (3). Upon completion of the reaction, hemin is added, resulting in peroxidase-like G4-hemin complex (4). Addition of tyramine causes the formed G4-hemine complex to generate a fluorescent signal using H2O2. The fluorescence intensity allows quantitative detection of exosomes (90, 91). An et al. investigated the short-term changes in exosomal protein levels before treatment, after chemotherapy (gemcitabine-based chemotherapy), and in the middle of chemoradiotherapy in locally advanced pancreatic cancer to find potential biomarkers of response to treatment. They identified eight proteins whose expression changed during therapy in all patients, possibly reflecting changes in expression levels in releasing tumor cells and could be potential biomarkers of treatment response or pancreatic cancer metastasis. Treatment downregulated the expression of 1B51 (B-51 alpha chain), Vimentin, and LYSC (endoproteinase Lys-C), whereas CD71 (transferrin receptor protein 1), MARE2 (microtubule-associated protein RP/EB family member 2)/APC-binding protein EB2, PARVB (parvin beta), PLF4 (platelet factor 4), and OBSL1 (obscuring-like protein 1) were upregulated in patient serum. Two of them, OBSL1 and PLF4, showed the strongest response to therapy in their dataset, and the researchers believe that these proteins are promising candidates for biomarker development (92). Melo et al. found that pancreatic cancer patients have a greater proportion of Glypican-1 (GPC1) expressing exosomes (100%) than healthy individuals (average 2.3%). In particular, they reported that this marker has excellent sensitivity and specificity in the early stages of pancreatic cancer (93). Similarly, EGFR is more abundant in the exosomes of gastric cancer patients, making it a potential diagnostic molecule (59). In a study of tumor-derived exosomes in the serum of glioblastoma patients, tumor-specific EGFRvIII was detected in 7 of the serum exosomes from 25 patients (59, 94). These results suggest that exosomal biomarkers could be found in the bloodstream or other sources and used to assess cancer invasion and progression with higher sensitivity and specificity (2).

A pilot study showed the presence of two known prostate cancer biomarkers, PCA-3 and TMPRSS2: ERG, in exosomes isolated from patients’ urine as a potential diagnosis and monitoring of prostate cancer patient status (95). In another study, RNA was extracted from urinary exosomes from twenty patients with prostate cancer and nine healthy men. Then, microRNAs were analyzed using next generation sequencing (NGS). Results indicate downregulation of five microRNAs, including miR-34a-5p, miR-196a-5p, miR-143-3p, miR-501-3p and miR-92a-1-5p in exosomes from prostate cancer patients. Among them, miR-196a-5p and miR-501-3p suggested as promising diagnostic biomarkers for prostate cancer (96).

In a study by Hu et al (97), overexpression of exosomal lncRNAs, i.e. LNCV6_98390, LNCV6_98602, LNCV6_38772, LNCV_108266, LNCV6_116109, and LNCV6_84003 proposed as potential diagnostic biomarkers for colorectal cancer (98). In the context of diagnosing this type of cancer, Ma et al. identified numerous candidate targets with a miRNA–mRNA network with high connectivity contained 13 hub mRNAs and five hub exosomal miRNAs (mRNA: CBFB, CDH3, ETV4, FUT1, FOXQ1, GRIN2D, GCNT2, KIAA1549, KRT80, LZTS1, SPTBN2, SLC39A10, ZSWIM4; and exosomal miRNA: hsa-miR-29c, hsa-miR-126, hsa-miR-139, hsa-miR-141, and hsa-miR-423) as potential biomarkers with high diagnosis value for CRC. Their findings open the door to new colorectal cancer diagnostic and therapeutic approaches (99). In addition, exosomal circTUBGCP4, which induces vascular endothelial cell tipping to promote angiogenesis and cancer metastasis by activating the Akt signaling pathway by inhibiting miR-146b-3p, has recently been proposed by Chen et al. as being produced by CRC cells (100).

Noteworthy increase level of both proteins, G3BP and PIGR, has been reported in the exosomes of HCC patients. Additionally, exosomal levels of miR-21, miR-224, miR-210 and miR-93 were significantly higher in the serum of HCC patients compared to healthy controls, while miR-9-3p and miR-638 were significantly lower (101).

Although the number of potential biomarkers identified for various cancers is increasing dramatically, there is still a large gap between biomarker studies and clinical applications. This is due to numerous problems, such as the low concentration in human body fluids and the low stability of these potential biomarkers (102). In **Table 1**, we summarized exosome biomarkers with diagnostic applications in various cancers. Since its discovery, ultracentrifugation has been the most commonly used technique for the isolation of exosomes (2). There are alternative methods such as filtration, chromatography, and bead isolation; however, the main approach of purification is based on size, which is a disadvantage because some protein fragments become entrapped in urine or serum during the isolation procedure, which can affect results (132, 133).

**Table 1:** Exosome biomarkers in a variety of cancer types.

Cancer	Exosomal biomarker	Type of exosomal biomarker	Type of body fluid	Ref.
**Breast cancer**	Survivin and Survivin-ΔEx3 Fibronectin, FAK, EGFR, Periostin, HER2, Del-1 CD47 miR-101, miR-105, miR-181a, miR-372 and miR-373 ssODNs	Protein Protein Protein Nucleic acid Nucleic acid	Serum Plasma Blood Serum Plasma	(86) (87, 103–106) (107) (88, 108) (109)
**Melanoma**	MIA, MICA and S100B	Protein	Serum	(110, 111)
**Ovarian cancer**	Claudin-4 CD24, TGM2, U2AF1, U2AF2, and HNRHPU miR-21, miR-141, miR-200a, miR-200c, miR-200b, miR-203, miR-205 and miR-214 miR-21 miR-30a-5p	Protein Protein Nucleic acid Nucleic acid Nucleic acid	Plasma Ascitic Fluid Serum Blood Urine	(89, 112, 113–116)
**Prostate cancer**	PSMA and PCA3 miR-141 and miR-375; miR-107 and miR-574-3p miR-196a-5p, miR-501-3p, ERG, PCA3, and SPDEF (RNA)	Protein Nucleic acid Nucleic acid	Urine Serum and urine Urine	(95, 96, 117–120)
**Pancreatic cancer**	Vimentin Glypican-1 miR-1246, miR-4644, miR-3976 and miR-4306 PCA-3 mRNA	Protein Protein Nucleic acid Nucleic acid	Serum Blood Serum Urine	(92, 93, 121, 95)
**Colorectal cancer**	GPC1 CD147 circ-KLDHC10 LncUEGC1 and LncUEGC2	Protein Protein Nucleic acid Nucleic acid	Plasma Blood Serum Serum	(122–125)
**Gastric cancer**	EGFR LncRNA HOTTIP	Protein Nucleic acid	Medium Serum	(59, 126)
**Glioblastoma**	angiogenin, IL-6 and IL-8 EGFRvIII (mRNA) and LncRNA-HOTAIR miR-1247-3p	Protein Nucleic acid Nucleic acid	Medium Serum Serum	(127, 127, 128, 53)
**Lung cancer**	LG3BP and PIGR LRG1	Protein Protein	Blood Urine	(129, 130)
**Cholangiocarcinoma**	VNN1, CRP, FIBG, IGHA1, and A1AG1	Protein	Blood	(129)
**Hepatocellular** **carcinoma**	miR-18a, miR-221, miR-222, miR-224	Nucleic acid	Serum	(131)

## 6 Devices used to detect exosomes

**Figure 3:**
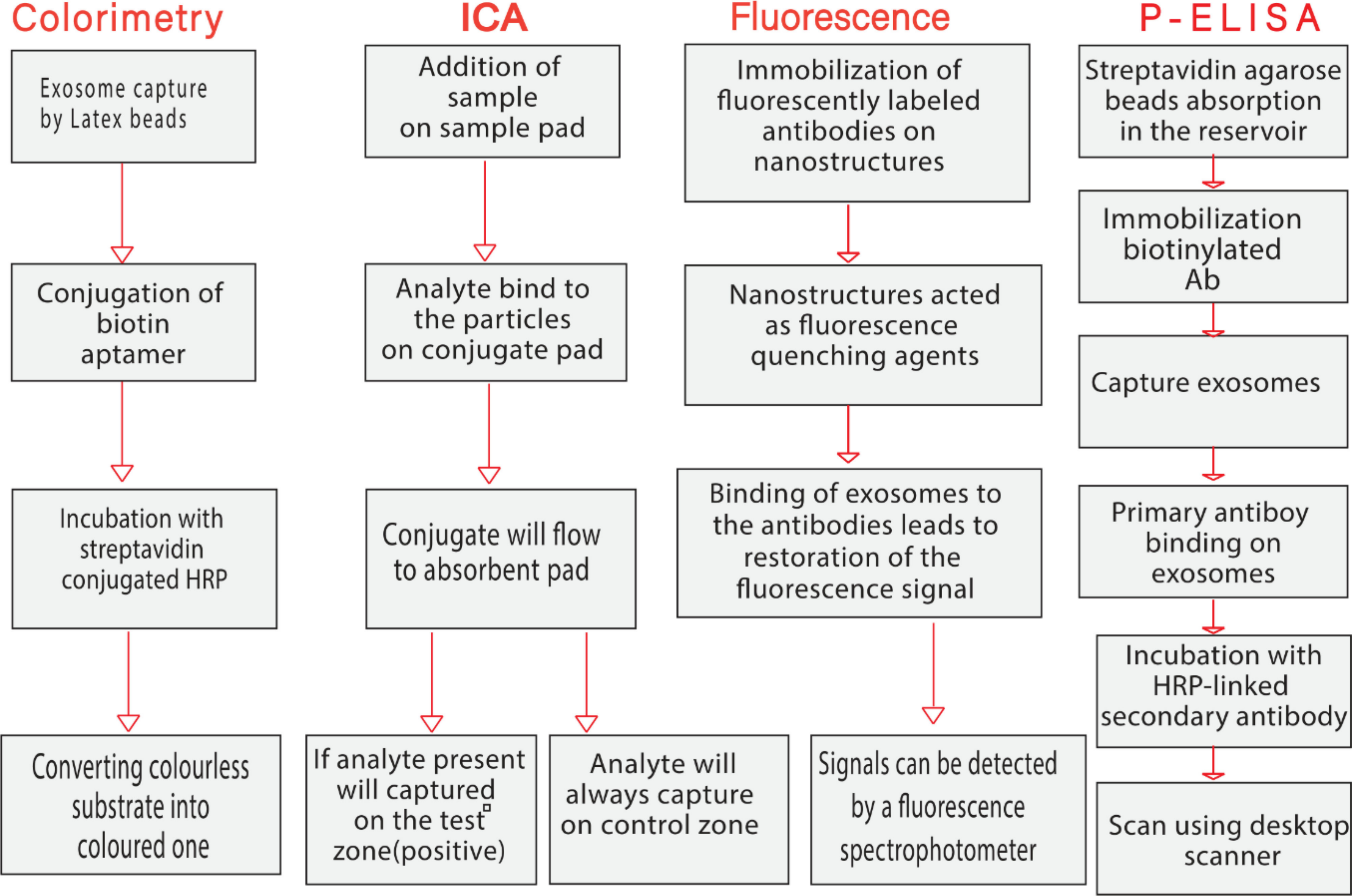
Schematic diagram representing the main steps of paper-based biosensors for isolation and measurement of circulating exosomes.

Exosomes and micro vesicles derived from cancer cells contain a wealth of proteomic and genetic data that can be used to predict cancer development, metastasis spread, and treatment efficacy. However, due to their small size (30 nm-1 nm), lengthy sample preparation required to isolate and measure them, they have not been widely used as biomarkers to improve patient care. Despite the growing interest in exosome research, there are still no effective quantitative methods for exosome identification. Exosomes in physiological fluids need to be detected and verified quickly and cost-effectively. Biosensors have attracted the interest of professionals and researchers around the world in recent years in the search for sensitive devices to detect exosomes (**Tables 2**, **3**) (143, 144). Biosensors contain a biological component, such as an enzyme, antibody, or nucleic acid, and a physicochemical transducer or transducing microsystem that converts the biological response into an electrical signal proportional to the intensity of an analyte in the response (3). Exosomes have been identified by a variety of methods in recent decades, including Western blotting (145), ELISA (146), and mass spectrometry (147). Despite their popularity and widespread use, these methods have drawbacks and limitations (7). Biosensors have several advantages over instrument-dependent technologies, including cost-effectiveness, ease of use, rapid response, high sensitivity, high specificity, and multiplexing capabilities (148). People in resource-poor settings who have not had access to modern diagnostic tools could also benefit from the development of biosensors for exosome detection, and cancer therapy could also be improved (149). Cancer-related antigens are abundant on the surface of cancer-derived exosomes (150). Therefore, exploiting variations in their surface composition is the most promising technique for rapid and easy identification of exosomes (3). Tetraspanins (such as CD9, CD63, CD81, and CD82) or lipid rafts (such as cholesterol, phosphatidylserine, and ceramide) on the surface of exosomes are commonly used as targets for exosome detection in biosensors (151). Depending on the cell type, a number of cancer-associated antigens such as CEA, EpCAM, HER2, IGFR, LMP1, MUC18, and PSMA are found on the surface of exosomes and are used as diagnostic and therapeutic markers for a variety of malignancies (152). The use of novel paper-based biosensors, such as colorimetry, fluorescence-based assays, immunochromatographic assays (ICA), and paper-based enzyme-linked immunosorbent assays (P-ELISA) to isolate and detect exosomes in clinical samples and their application in cancer monitoring and diagnosis are discussed in the next section (**Figure 3**).

**Table 2:** Summary of paper-based biosensors.

Method	Type of detection	Signal element	Recognition element	Target	Sample	Detection limit	Ref.
**Colorimetry**	Optical Optical Optical	Gold nanoparticles Gold nanoparticles Gold nanoparticle, carbon black, and magnetic nanoparticles	Antibody Antibody Antibody	Exosomal MICA and CD9 Exosomal CD9, CD81, and CD63 Exosomal CD9, CD81, and CD63	Melanoma (Ma-Mel- 55 and Ma-Mel-86c) cells and human serum Melanoma (Ma-Mel- 86c) cells and human Plasma Human plasma	5 × 10^7^ exosomes/µl 8.5 × 10^5^ exosomes/µl 3.4 × 10^6^ extracellular vesicles/µl	(111, 134, 134, 135) (134, 136)
**Fluorescent**	Optical	Upconversion nanoparticles (UCNPs) and gold nanorods (AuNRs)	Split aptamer	Exosomal CD63	Hepatocellular carcinoma (HepG2) cells	1.1 × 10^3^ particles/µl	(137)
**ICA**	Optical Optical	Gold nanoparticles Quantum dots	Antibody Antibody	Exosome biomarkers Exosome biomarkers	Breast tumor (MCF-7) Cells Oral squamous carcinoma (Cal27) cells	1.3 × 10^3^ particles/µl 2 × 10^3^ particles/µl	(14, 138)
**p-ELISA**	Optical	HRP-linked secondary antibody	Antibody	Exosomal CD9 and CD63	Lyophilized exosome standards and human serum	9.29 × 10^10^ particles/ml	(139)

**Table 3:** The advantages and disadvantages of different paper-based biosensors.

Method	Advantages	Disadvantages	Ref.
**Colorimetry**	Easy-to-use detection Rapid response Naked-eye detection Cost-effective system	Low sensitivity Limited multiplexing capability Limited quantification capability	(3)
**Fluorescent**	High sensitivity Flexible fluorescence-quenching Capability Multiplexing capability	Costly equipment with both excitation light and fluorescence measurement Fluorescent label may impair binding	(3)
**ICA**	Simplicity Easy operation Low-cost Portability	Low sensitivity	(140)
**p-ELISA**	Fast Easy to use No special equipment required Sample efficiency	Separate exosomes with targeted proteins only	(141, 142)

### 6.1 Colorimetry

Colorimetric assays use a visible signaling agent to monitor target molecules and detect the absence or presence, as well as the concentration, of the analyte of interest through the evaluation of color production or color change. Colorimetric tests measure changes in absorbance or reflectance intensity caused by chemical or biochemical reactions between the target and chromogenic probes. Colorimetric methods typically provide qualitative measurements with visual responses, but color analysis on smart devices can convert them to quantitative measurements. They are now viewed as very promising tools in the diagnosis of diseases like cancer because of their quick and simple integration with technological devices (153).

Colorimetric biosensors are often visible to the naked eye or *via* simple handheld optical detectors as a color change that provides a yes/no answer or a semi-quantitative result without additional analytical equipment (3). Colorimetric biosensors are divided into two types: paper-based and solution-based. Paper-based colorimetric biosensors, which use paper as a substrate, are ideal for field use because they are easy to use and require a minimal amount of sample (3). Solution-based colorimetric biosensors, which typically use Au-NPs or enzymes as signal markers to produce a color shift as a colorimetric signal, could be advantageous for rapid and easy detection with relatively large sample volumes (3). Xu et al. developed a sensitive and selective colorimetric aptasensor for the detection of cancer exosomes enabled by horseradish peroxidase (HRP)-accelerated dopamine polymerization and dark-stained polydopamine (PDA) *in situ* deposition. Target exosomes were first captured by latex beads through aldimine condensation, followed by biological recognition using a specific CD63/LAMP-3 aptamer conjugated to HRP through biotin-streptavidin binding. The color intensity correlated with the amount of LAMP-3 and the detection limit was only 7.7×10^3^ particles/ml, which is an improvement of 3-5 orders of magnitude over conventional dot-blot methods (154).

The research by Yu and colleagues (155) consists of an LFIA built on a competitive strategy to find exosomes derived from non-small lung cancer (NSCLC). Because aptamers have advantages over Abs, such as high affinity and stability, they were used as a biorecognition component against the CD63 protein on the exosome membrane. In order to compete with the AuNPs functionalized with CD63 aptamer, by a thiol-gold functionalization strategy, that were used as colorimetric probes, a streptavidin-biotin-CD63 aptamer was immobilized on the NC membrane. The amount of exosomes used in this assay is inversely correlated with the color seen at the test line. The LOD of A549 exosomes, which were isolated from human lung carcinoma cells, was 6.4 × 10^9^ particles/mL.

### 6.2 Immunochromatographic assay

Immunochromatographic assay (ICA), namely Lateral flow biosensors (LFBs) are novel paper-based devices that are considered one of the most promising new technologies due to their simplicity, rapid analysis, low cost, and high sensitivity and specificity (140). The combination of immunochemical reactions and chromatography- the separation of sample components based on differences in the way they move through a sorbent- is the basic idea of LFB. LFBs typically consist of four parts: a sample pad, a conjugate pad, a nitrocellulose membrane with test and control sections, and an absorbent pad. In general, the sample solution migrates across the pads due to capillary forces. In a sandwich format, NPs-labeled protein–target complexes and free capture antibodies accumulate in two defined zones comprising the test and control areas, respectively. Accumulation of NPs in the test section results in a color change that can be detected based on the concentration of NPs in the test section. The major advantage over other immunoassays is that the entire test can generally be performed in a single step and in a few minutes (3). The “lock and key” concept of antigen-antibody interaction is the best-known biological recognition (156, 157). Exosomal proteins are effective antigens, thus antigen-antibody immunoreactions are well-established in biosensor production (135, 136). Various nanomaterials have been explored as label for the LFB strips including colloidal AuNPs, UCNPs (upconverting nanoparticles), and, QDs (quantum dots) to improve the assay sensitivity and visualize the interactions between targets and receptors (140). The construction of optical biosensors based on nanomaterials for target sensing is an ongoing and unique trend in the field of analytical diagnostics (158). Colloidal AuNPs are the most commonly used nanomaterials in ICA/LFB. Wu and colleagues developed a dual AuNPs biosensor for rapid detection and molecular profiling of exosomes using a LFB. CD9/MIC3 antibodies capable of targeting exosomes were integrated into 15 nm AuNPs. To generate signal amplification, 40 nm AuNPs were used to bind to 15 nm AuNPs. The colorimetric biosensor proved suitable in fetal bovine serum and was able to rapidly detect exosomes from MCF-7 cells with a limit of detection (LOD) of 1.3×10^3^ particles/µL (more than 12-fold compared with the normal lateral flow assay), and there was a linear relationship between the intensity of the test zone and exosome concentrations ranging from 7.4×10^3^ to 5.55×10^4^ particles/μL (14).

### 6.3 Fluorescent

Based on the analyte concentration on the sensing surface, the fluorescence methodology emits light. The fluorescence label transitions from the fundamental energy level to a higher energy level when excited by a beam of light. As a result of the molecule’s instability at this energy level, it emits the extra energy it took in as light emission and descends to the fundamental energy level. The fluorescence signal against the analyte amount is found by measuring the light emission during this change in energy level (159).

Fluorescent biosensors designed for exosome identification have significant advantages due to their high sensitivity, good selectivity, and ease of use (7). Typically, fluorescent dyes, fluorophores, fluorescent proteins, or fluorescent nanoparticles are used to generate a fluorescent signal (3, 7). Fluorescent biosensor systems are classified into three types: paper-based, solution-based, and microplatform-based. Paper-based fluorescence biosensors, also known as paper-based analytical devices (PADs), are a basic analysis platform that can be built by oneself (137). These biosensors provide an accurate and versatile method for detecting exosomes in a variety of instruments (160). In the presence of a fluorescent molecule and a quencher, the system can produce a fluorescent “on” or “off” signal. The main concept is to combine the fluorescence detection with the high specificity of ligand-binding proteins. For example, a fluorometric biosensor was developed to detect exosomes produced by breast cancer cells. In the detection method, fluorescently labeled anti-CD63 was immobilized on the surface of molybdenum disulfide-multiwall carbon nanotubes (MoS2-MWCNT), which acted as fluorescence quenching agents due to the fluorescence resonance energy transfer (FRET) or electronic energy transfer (EET) from PE-conjugated antibody to the MoS2-MWCNT nanostructure. When the exosomes were bound to the anti-CD63 antibodies, the fluorescence recovered and the strength was increased by increasing the number of exosomes (161). Fluorescence can be used in combination with a variety of signal amplification techniques to create sensitive biosensors for exosome detection. Li et al. used Zr4+ as a bridge to join exosomes and liposomes because of the intrinsic combination of Zr4+ with phospholipids in both exosomes and liposomes. Due to this interaction, exosomes could be easily detected without complicated procedures (162).

### 6.4 Paper-based enzyme-linked immunosorbent assays

Immunological separation techniques can accurately collect EVs/exosomes *via* immunoaffinity (163, 164). The technique-based enzyme-linked immunosorbent assay (ELISA) for EVs/exosomes, first published by Logozzi and colleagues in 2009, enables the collection, detection, characterization, and quantification of extracellular vesicles in human body fluids and supernatants from cultured cells. The paper-based ELISA (p-ELISA) is faster and less expensive than conventional ELISA. It provides the same level of sensitivity and specificity, but requires lower reagent and sample volumes and processing time, resulting in lower analytical costs (165). p-ELISA is a powerful platform that meets the criteria set by the World Health Organization ASSURED (affordable, sensitive, specific, user-friendly, rapid and robust, instrument-free, and available to end users) for disease diagnostics in resource-limited regions/countries. However, the p-ELISA has low sensitivity and linearity (139). Lee et al. developed p-ELISA for targeted detection and separation of exosomes using streptavidin agarose resin-based immobilization (SARBI). This technique shortens assay preparation time and provides strong binding (due to the high affinity of biotinylated Ab and streptavidin-agarose). They studied SARBI p-ELISA systems with and without CD63 capture-AB immobilization, as well as with fetal bovine serum (FBS) and EVs/exosome-depleted FBS (dFBS). CD63 is a protein found on the membranes of many different cell types. The results demonstrate the capabilities of SARBI p-ELISA and its high sensitivity and linearity compared to other ELISA techniques (139).

## 7 Paper-based biosensors as effective medical diagnostic

The production of accurate, easy-to-use, and cost-effective biosensors for protein detection is of great interest to clinical diagnostics (166). Paper-based biosensors could be a solution to these needs. Namely, they are inexpensive and abundant; sensitive because they can be based on immunoreactions or nucleic acid hybridizations; user-friendly because the pregnancy test is one of the most commonly used POC biosensors; rapid because the reaction takes only a few minutes or less; Instrument-free because they can be observed with (or visible to) the naked eyes; and small and affordable when quantitative detection is required (166).

Paper-based biosensors are divided into three types: Dipstick assays, microfluidic paper analyzers (µPADs), and lateral flow assays (cs). Dipstick assays are the most popular and simplest type of commercially produced paper-based diagnostics. They are commonly used for periodic qualitative testing of urine samples on a strip of pH paper to determine the value of PH (167). Oliveira-Rodríguez et al. developed a colorimetric lateral flow immunoassay (LFIA) for the accurate quantification of exosomes from a human metastatic melanoma cell line based on tetraspanins (CD9, CD63, and CD81) as enriched exosome biomarkers in the membrane of exosomes (135). In this study, LFIA was performed in dipstick format, and tetraspanins were used as targets for antibody capture and detection because they are widely distributed in exosomes of all cell types. For dipstick analysis, exosomes are embedded in the sample between a mixture of anti-CD9 and anti-CD81 (1:1) as immobilized antibodies on the assay section for visualization of exosomes from different sources and gold nanoparticle-conjugated anti-CD63 as detection antibody (135). The unbound AuNP conjugates migrated forward where they were captured by anti-mouse immunoglobulin antibody (control, C) to verify the function of the system (135).

μPADs are a new class of analytical instruments based on cellulose materials that can be used to analyze complex biological samples such as macromolecules, proteins, nucleic acids, toxins, microbes, and diseases. µPADs have been developed to overcome many barriers and even eliminate standard detection methods. In addition, they are relatively inexpensive and do not require a pump or other external energy source to move the fluid through the channel. They also require a very small sample volume and can be used for multiplex and quantitative analyzes (168). Lateral flow assay strips (LFAs), like dipsticks, have all the chemicals upstream on the strip, but they also contain the sample flow. The liquid sample containing the analyte of interest moves through the different zones of the strip without the aid of external forces (capillary action) associated with molecules that may interact with the analyte. Many other assay designs, such as sandwich and competitive formats or multi-detection, can be used in this manner (166, 169). Several paper-based sensors have been constructed using different methods (166). The sandwich assay based on a pair of antibodies (immunosandwich formation) is the most common format used in LFAs-based paper biosensors for the detection of proteins, as described below. Using a portable fluorescence biosensor with quantum dots and a LFAs; Li et al. identified nitrated ceruloplasmin, an important biomarker for cardiovascular disease, lung cancer, and stress responses to smoking. They integrated quantum dot signals into a lateral flow assay strip, resulting in high sensitivity, selectivity, and speed of protein identification. In a spiked human plasma sample, the researchers achieved a LoD of 8 ng/mL (170). Lin et al. also used the sandwich format for rapid and sensitive detection of prostate-specific antigen (PSA) in human serum. They presented nanoparticles (NPs) coated with anti-PSA antibodies and labeled with IEB (immunochromatographic electrochemical biosensor) to fuse the immunochromatographic strip with the electrochemical detector for quantitative signal transduction (171). For signal amplification, the anti-PSA antibodies were labeled with CdSe/ZnS. The sandwich immunoreaction was performed on an immunochromatographic strip, and the electrochemical signals depend on the amount of anti-PSA QDs conjugates adsorbed on the contact zone. The results showed that simple, accurate, sensitive (with a detection limit of 0.02 ng mL1 PSA), reproducible (with a relative standard deviation of 6.4%), and less costly PSA detection in human serum is possible (171). Dong et al. developed a simple, rapid method to quantify EVs by combining a membrane biotinylation technique and a fluorescent nanosphere (FN) based lateral flow assay. Based on the common property with phospholipid membranes, the lipid membrane of EVs can be successfully modified with biotin, efficiently and selectively with biotin-functionalized phosphatidylethanolamine (DSPE-PEG-biotin). Subsequently, a lateral flow assay using FNs as reporters was used to obtain quantification based on the strong affinity between streptavidin and biotin. Biotinylation of biogenic EVs can be achieved at a rate of 85%. Using the proposed approach, 2.0×10^3^ particles can be detected. The entire operation took less than an hour. This method has also been used to identify EVs in biological samples, indicating potential clinical applications (138). Xu et al. used aptamer-functionalized gold nanoparticles (AuNPs) to construct a lateral flow biosensor, also known as a dry-reagent strip biosensor, to detect thrombin in human plasma. They demonstrated that an aptamer-based dry-reagent strip biosensor could detect thrombin in human plasma samples with equal sensitivity and even greater specificity than an antibody-based strip biosensor (172). The biosensor response was linear over a thrombin concentration range of 5-100 nM, with a LoD of 2.5 nM.

The detection of nucleic acids is used not only for genetic testing but also for the detection of pathogens, which gives these molecules enormous importance in diagnostics (173). Samples are usually preamplified by PCR or isothermal methods to ensure sufficient DNA for detection, or are synthetically produced DNA sequences (166). In addition, coupling of DNA sequences to paper is often achieved by using a protein pair, such as biotin-avidin or antigen-antibody, to provide a link between the DNA and the paper. This opens a wide range of possibilities for the use of aptamer-functionalized gold nanoparticle probes in dry reagent strip biosensors for protein detection at the point-of-care or in the field. He et al. developed an ultrasensitive nucleic acid biosensor (NAB) based on a lateral flow strip biosensor(LFSB)and dual labels of HRP and gold nanoparticles (Au-NP) (174). The combination of the unique optical properties of Au-NPs with the deposition of an insoluble enzymatic catalyst product provides a dramatic ability to detect 0.01-pM target DNA, allowing for rapid, sensitive, and inexpensive detection without specialized equipment (174).

Electrochemical or optical transducers can detect the red chromogen product. Compared to standard hybridization assays, NAB provides a rapid, sensitive, and inexpensive technique for detecting nucleic acid samples. This technique has significant potential for on-site diagnosis of genetic diseases and identification of infectious agents (174). Konstantou et al. used a dipstick dry reagent assay to detect the somatic mutation JAK2V617F, which is associated with polycythemia vera and thrombocythemia and has been included as a clonal biomarker in the diagnostic criteria of WHO (174). The method uses a triprimer PCR (one forward and two reverse reactions) with visual detection of reaction products by the naked eye within minutes, without the need for special equipment or multiple pipetting and incubation steps (174). Researchers used biotin- or digoxin-modified primers in a triprimer PCR to detect a single nucleotide polymorphism (SNP) in clinical samples. In fact, the digoxin primer matches only the mutant allele, resulting in a short product, whereas the biotinylated primer always matches, resulting in a long product. The specificity of the dipstick assay results from both the PCR step and hybridization with probes specific for the long and short products. Both products are further hybridized with dA probes before being pipetted onto the sample pad and identified by AuNPs modified with oligo dT. Streptavidin is used as a control line, while an antibody specific for digoxin is used as a detection line. The dipstick dry reagent assay provides a rapid and specific method for detecting the JAK2V617F allele in a sample without the need for specialized equipment or highly skilled technical personnel (175). He et al. established a simple and accurate method for visual identification of R156 mutations (R156H and R156C) of keratin 10 in epidermolytic hyperkeratosis based on isothermal strand displacement polymerase reactions (ISDPR) and lateral flow strips (LFS) (174). Epidermolysis hyperkeratosis (EHK) which is also called Bullous congenital ichthyosiform erythroderma (BCIE), is an autosomal dominant skin disease caused by mutations in the keratin 1 (KRT 1) or keratin 10 (KRT 10) genes. The principle of visual detection of the keratin 10 mutation was to design a series of biotin-modified hairpin and digoxin-modified primers for ISDPR that have complementary sequence to the mutant DNA. The R156H-mutated DNA resulted in ISDPR generating numerous biotin- and digoxin-linked duplex DNA products in the presence of biotin-modified hairpin DNA and digoxin-modified primer. Two immunoreactions were used to visually identify the generated duplex DNA complexes on an LFS (the digoxin-anti digoxin-labelled Au-NPs on the conjugate pad and the biotin-anti biotin antibody reaction on the test zone of the LFS). The concentration of Au-NPs on the test zone leads to the detection of the mutant gene without instrumentation. The LoD reaches up to 1 FM (176). Paper-based surface-enhanced Raman spectroscopy (SERS)-vertical flow biosensor, named iREX (integrated Raman spectroscopic EXO) biosensor, for multiplexed quantitative profiling of exosomal proteins in clinical serum samples of patients. This biosensor used to quantitatively profile HER2, MUC1, and CEA in EXO samples derived from different breast cancer cell subtypes. It seems that the iREX biosensor with high sensitivity, powerful multiplexing capability, molecular specificity, and high diagnostic accuracy could be a promising clinical tool for personalized diagnosis and precise management of patients with breast cancer (177).

Paper-based biosensors can also be integrated with electrochemical biosensors (i.e., amperometric or voltammetric biosensors) by printing multiplexed sensor electrodes directly on paper. Paper with multiplexed sensor electrodes can simultaneously detect multiple analytes down to the picogram-per-milliliter level (178).

## 8 Conclusion

The exosomes has emerged as a potential biomarker for cancer detection. Therefore, there is a great need for simple, affordable, and user-friendly biosensors for rapid, accurate, and quantitative detection of exosomes. This review summarizes important aspects of exosomes in cancer development and their potential as diagnostic biomarkers. We also reviewed the recent advances, characteristics, advantages, and limitations of different types of paper-based biosensors (dipstick, lateral flow assays, and paper-based microfluidic analyzers) for the isolation and analysis of cancer exosomes. These results suggest that paper-based biosensors for exosome detection are becoming a potential alternative to conventional approaches in POC diagnosis and prognosis, as they offer advantages such as short detection time and low cost. However, research in exosome detection is still at an early stage, and many problems and critical issues in the field need to be resolved before they can be used in clinical applications. These challenges include improving the stability of exosome analysis, identifying exosomes secreted by almost all somatic cells, the need for large sample volume, and challenges associated with handling clinical samples. Overcoming each of these challenges will have a critical impact on the development of biosensors for exosome detection.

## Author contributions

HM involved in conception, design, and drafting of the manuscript. HH, MM, MK, BK contributed in data collection and manuscript drafting. All authors contributed to the article and approved the submitted version.
